# Achieving consensus on priority items for paediatric palliative care outcome measurement: Results from a modified Delphi survey, engagement with a children’s research involvement group and expert item generation

**DOI:** 10.1177/02692163231205126

**Published:** 2023-10-18

**Authors:** Lucy Coombes, Daney Harðardóttir, Debbie Braybrook, Hannah May Scott, Katherine Bristowe, Clare Ellis-Smith, Lorna K Fraser, Julia Downing, Myra Bluebond-Langner, Fliss EM Murtagh, Richard Harding

**Affiliations:** 1 King’s College London, Florence Nightingale Faculty of Nursing Midwifery and Palliative Care, Cicely Saunders Institute, London, UK; 2 Royal Marsden NHS Foundation Trust, Sutton, UK; 3 International Children’s Palliative Care Network, Kampala, Uganda; 4 University College London, Louis Dundas Centre for Children’s Palliative Care, London, UK; 5 Rutgers University, Camden, NJ, USA; 6 Wolfson Palliative Care Research Centre, Hull York Medical School, University of Hull, Hull, UK

**Keywords:** Outcome assessment, Delphi survey, public participation, palliative care, children

## Abstract

**Background:**

There is no validated outcome measure for use in children’s palliative care outside sub-Saharan Africa. Stakeholders must be involved in the development of such measures to ensure face and content validity.

**Aim:**

To gain expert stakeholder consensus on items for inclusion in a paediatric palliative care outcome measure to establish face and content validity.

**Design:**

This study was conducted in two phases following Rothrock and COSMIN guidance on patient-reported outcome measure development. Phase 1: Three-round modified Delphi survey to establish consensus on priority items. Phase 2: Item generation meeting with key stakeholders to develop initial measure versions. A young person’s advisory group was also consulted on priority outcomes.

**Setting and participants:**

Delphi survey: Parents and professionals with experience of caring for a child with a life-limiting condition. Young person’s advisory group: young people age 10–20 years. Item generation meeting: bereaved parents, academics and clinicians.

**Results:**

Phase 1: Delphi survey (*n* = 82). Agreement increased from Kendall’s *W* = 0.17 to *W* = 0.61, indicating movement towards consensus. Agreement between professional and parent ranking was poor (Cohen’s kappa 0.13). Professionals prioritised physical symptoms, whereas parents prioritised psychosocial and practical concerns. Advisory group: Children (*n* = 22) prioritised items related to living a ‘normal life’ in addition to items prioritised by adult participants. Phase 2: Five age/developmental stage appropriate child and proxy-reported versions of C-POS, containing 13 items, were drafted.

**Conclusions:**

This study highlights the importance and feasibility of involving key stakeholders in PROM item generation, as important differences were found in the priority outcomes identified by children, parents and professionals.

**What is already known about this topic?**

- Children and young people with life-limiting and life-threatening conditions experience many inter-related symptoms, concerns and care priorities that require a holistic approach to care.
- There is currently no validated patient-centred outcome measure (PCOM) for use in paediatric palliative care outside of sub-Saharan Africa.
- Development of such a measure has repeatedly been highlighted as a clinical and research priority.

**What this paper adds?**

- This study describes the item generation phase of the development of a novel PCOM with demonstrated face and content validity for use in paediatric palliative care (C-POS).
- Involvement of key stakeholders in item generation has demonstrated important differences in the priority healthcare outcomes identified by children, parents and healthcare professionals in paediatric palliative care.
- Five versions of C-POS have been developed that reflect variation in age/developmental stages of the target population and allow for proxy reporting if required.

**Implications for practice, theory or policy**

- A PCOM that considers psychosocial domains will support professionals to assess needs more holistically.
- Further research is required to test C-POS cognitively and psychometrically prior to implementation.

## Background

It is estimated that each year 21 million children and young people worldwide (hereafter ‘children’) with life-limiting or life-threatening (‘life-limiting’) conditions require input from palliative care services.^1^ Life-limiting conditions are those for which there is no hope of cure, and from which children will die. Life-threatening conditions are those for which curative treatment may be feasible, but may fail.^2^ With advances in medical care, increasing numbers of children are living longer with life-limiting conditions.^3,4^ Provision of children’s palliative care varies geographically, and increased prevalence of life-limiting conditions has not been met with an equivalent increase in healthcare resource allocation.^3,5^ Children with life-limiting conditions experience a multitude of inter-related symptoms, concerns and care priorities that impact on all aspects of daily life.^6^ This requires a holistic, child-centred approach to care.

A patient-reported outcome measure (PROM) is defined as a measure of a patient’s health status, elicited directly from the patient. Many palliative care patients, including children with life-limiting conditions, are too unwell or cognitively unable to self-report on their own health outcomes.^7^ A measure which allows for proxy completion is required. Together PROMs and proxy-reported measures are termed patient-centred outcome measures (PCOMs).^7,8^ The use of PCOMs in adult palliative care has been shown to improve service quality and promote patient-centred care,^9^ as well as lead to better symptom recognition, more discussion of quality of life and increased palliative care referrals.^7^ PCOMs have been advocated for improving awareness of unmet need, understanding different models of care delivery and allowing national and international comparison.^10,11^

Evidence of the use of PCOMs in paediatric palliative care is lacking due to absence of a validated measure.^12^ Development of a PCOM for use in this population has been repeatedly highlighted as a priority.^13–17^ A psychometrically validated measure exists in sub-saharan Africa (recently adapted in Belgium) where the sample informing content validity predominantly had a HIV diagnosis^18,19^ This measure was developed before current PCOM development guidance had been established.^20,21^ The Belgian version has undergone initial face and content validation but further psychometric data is not available.^22^

This study is part of a programme of work to develop the Children’s Palliative Outcome Scale (C-POS), a child-centred outcome measure for use in paediatric palliative care. This measure is being developed within the UK healthcare context, with parallel processes to develop C-POS in other regions. Previous sequential outputs are two systematic reviews (establishing the need for a new PCOM,^12^ identifying response formats and administration modes used in PCOMs for children^23,24^) and primary qualitative data identifying symptoms, concerns and care priorities (the sample included children and young people, health and social care professionals, siblings, parents and commissioners).^6,25^ This previous work has demonstrated that several versions of C-POS will be required to reflect the age/developmental stages of children with life-limiting conditions. The aims of the study presented here were to: gain expert stakeholder consensus on items to be included in C-POS; further enhance face and content validity and finalise initial versions of C-POS for cognitive testing.

## Methods

**Figure 1.**
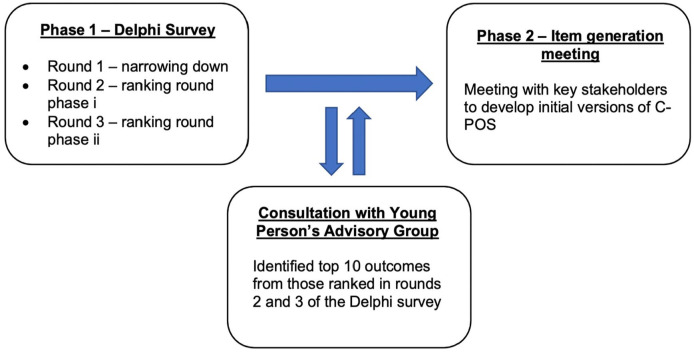
Study flow diagram.

C-POS is being developed following the Consensus-based Standards for the selection of health Measurement Instruments (COSMIN) and Rothrock guidance on PROM development.^21,26,27^ This paper reports on a Delphi survey, engagement with a young person’s advisory group, and an item generation meeting. A flow chart of the study is shown in Figure 1.

### Phase 1 – Delphi Survey

#### Study design

A modified Delphi ranking survey was conducted and reported in accordance with CREDES, between November 2020 and February 2021.^28^ A typical ranking Delphi survey has three phases: a) ‘brainstorming’ – experts list items important for the area of interest, b) ‘narrowing down’ – items identified in step one are narrowed down and c) ‘ranking’ – experts rank the remaining items over multiple rounds, with the aim of reaching consensus.^29,30^ Our previous work identifying symptoms, concerns and care priorities for children with life-limiting conditions and their families served as the ‘brain-storming’ phase.^6,25^ This paper reports on the ‘narrowing down’ and ‘ranking’ phases conducted using SmartSurvey^TM^.

#### Study procedure

COSMIN guidance on PROM development states that experts (including patients) should be included in measure development to ensure face and content validity.^20^ We included parents/carers (‘parents’) of children with life-limiting conditions as experts, and health and social care professionals (‘professionals’) to enhance validity and ensure clinical relevance.

*Eligibility criteria* Professionals with >6 months experience of caring for children with life-limiting conditions; parents of children 0 > 18 years with a life-limiting condition; bereaved parents whose child (0 > 18 years) had died of a life-limiting condition 12–24 months prior to consenting to participate.

*Recruitment* Professionals were recruited via the Association of Paediatric Palliative Medicine (UK doctors, nurses and allied health professionals), social media (UK paediatric palliative care charities, and researcher and institute Twitter pages) and clinical members of the study steering group.^6^ Parents were recruited via a UK a children’s palliative care charity, parents’ groups and social media.

#### Data collection

##### Round 1-‘Narrowing down’

The 42 outcomes identified from our previous work were presented in random order to each participant.^6^ Participants were asked to select the 20 items most important for inclusion in C-POS, and to suggest any items they thought were missing. A free text box allowed participants to explain their choices.

##### Rounds 2–3-‘Ranking’

Participants from the previous rounds were presented with the results in plain English terms. Participants were asked to rank the outcomes retained from round 1 in order of priority for inclusion in C-POS from most to least important. Items were presented in random order for the first ranking round and according to mean rank in subsequent rounds.^30^ A free text box allowed participants to explain their rankings. Weekly reminder emails were sent to those who had not responded. Each round was open for 2–3 weeks.

#### Data analysis

##### Round 1-‘Narrowing down’

Items selected by >50% of participants were moved to the ranking rounds.^30^ Data were analysed as a whole group, and separately for professionals and parents. New suggested items were compared with existing items and discussed by the research team and study steering group to gain expert consensus on whether they should be included in round two.^31,32^ The study steering group comprises parents whose child had died of a life-limiting condition, academics with expertise in PROM development, and professionals who care for children with life-limiting conditions. The steering group is responsible for reviewing the progress, quality and delivery of the C-POS study.

##### Rounds 2–3-’Ranking’

Kendall’s *W* coefficient of concordance and top half rank (percentage of participants who ranked items in their top 50%). Kendall’s W was interpreted as follows: weak < 0.5, moderate 0.5–0.7, strong>0.7.^29^ Cohen’s kappa was used to determine agreement between parent and professional rankings.

##### Stopping criteria

Data were analysed as per the previous round. If consensus was reached (Kendall’s *W* > 0.7) then no further rounds would be undertaken.

Data analysis was conducted using Stata (v16, StataCorp LLC, College Station, TX).

#### Ethics and consent

Ethical approval was obtained from King’s College London (MRSP-19/20-18826). Participants received written study information and completed a consent form at the beginning of each round.

### Consultation with Young People’s Advisory Group

The research team worked with an existing young person’s advisory group at a UK tertiary children’s hospital. The group comprised children and young people aged 10–21 years with a life-limiting condition, siblings of children with life-limiting conditions or those interested in a career in healthcare or research. During a virtual advisory group meeting in March 2021 the group were given a short, age-appropriate presentation on the C-POS study aims and some simple definitions of outcome measures and life-limiting conditions. The group was then divided in two by age. Older representatives were asked to work independently to review outcomes from those ranked during rounds two and three of the Delphi and choose their top 10 (Table 3). Younger representatives were asked to choose their top ten outcomes from this list as a group. Both groups were also asked to suggest names for the C-POS versions (as age bands to label measures is not appropriate in this population given common developmental delay). The groups facilitators led the session with support from a member of the research team. The intention was that working with the advisory group would strengthen and broaden the perspectives of children in the study and ensure children’s views continued to be considered in measure design.

Representatives were providing patient and public involvement and thus ethical approval was not required.^33^ Involvement is reported in line with GRIPP2 (short-form) guidance.^34^

### Phase 2 – Item generation meeting

This consisted of a half-day virtual meeting with the C-POS steering group. The agenda was informed by previous PROM item generation meetings.^35^ The meeting began with a presentation from the research team including: an overview of the study and the results from previous development work.^6,23,25^ the Delphi survey, and findings on aspects of measure design (recall period, response format, administration mode) from our qualitative interviews. Discussion was led by the research team, starting with the construct to be measured and the corresponding overarching themes found in our interview study (physical symptoms, spiritual/existential, social/practical and emotional/psychological), followed by suggestions on potential wording of questions. Also discussed were priority items for inclusion and aspects of measure design. After the item generation meeting, versions of C-POS were drafted for future cognitive and psychometric testing.

## Results

### Phase 1 Delphi survey

#### Round 1 – narrowing down

Eighty-two individuals participated (59 healthcare professionals, 23 parents/carers (one bereaved)). See Table 1.

**Table 1.** Participant demographics: Delphi round 1 – ‘narrowing down’.

Health and social care professionals (*n* = 59)		Parent/carers (*n* = 23)	
Gender (male:female)	8:50 (1 preferred not to answer)	Gender (male:female)	0:23
Profession	4 Counsellor/therapist 16 Doctor 4 Health care assistant 32 Nurse 1 Physiotherapist 2 Social work	Child’s diagnosis	1 Cancer 3 Circulatory 5 Congenital 2 Genitourinary 4 Metabolic 8 Neurological
Place of work	5 Community 30 Hospice 17 Hospital 7 Multiple settings	Child’s age in years (mean; range)	8.9 (1–17)
Experience in years (mean; range)	11.8; (1–30)	Ethnic background	4 mixed ethnic group 23 white British (parent/carer) 19 white British (child)

Twenty-one outcomes were selected by >50% of participants. Two additional outcomes were selected by >50% of the professional group, and three by the parent/carer group (Table 2). Twenty-three suggestions were made for additional outcomes. Most suggestions were thought to be incorporated in existing outcomes, except for one regarding siblings (suggested by 22% of parent participants).

**Table 2.** Results Delphi round 1 – ‘narrowing down’.

Outcome	Overall (*n* = 82)	Parent/carer (*n* = 23)	HSCPs (*n* = 59)
	n(%)	n(%)	n(%)
Pain^a^	73 (89.0)	18 (78.3)	55 (93.2)
Having sufficient support from health and social care professionals^a^	70 (85.4)	19 (82.6)	51 (86.4)
Reducing the impact of illness on family life/burden of care^a^	68 (82.9)	22 (95.7)	46 (78.0)
Child being able to do things they enjoy^a^	68 (82.9)	22 (95.7)	46 (78.0)
Ability to live life to the fullest^a^	67 (81.7)	22 (95.7)	45 (76.3)
Breathing and respiratory difficulties^a^	63 (76.8)	14 (60.9)	49 (83.1)
Tiredness or fatigue^a^	62 (75.6)	19 (82.6)	43 (72.9)
Emotional impact of illness^a^	59 (72.0)	20 (87.0)	39 (66.1)
Being able to maintain relationships with peers^a^	59 (72.0)	19 (82.6)	40 (67.8)
Being supported/enabled to express emotions and feelings^a^	57 (69.5)	17 (73.9)	40 (67.8)
Having a plan for future care^a^	55 (67.1)	19 (82.6)	36 (61.0)
Being able to take part in memory making opportunities^a^	54 (65.9)	19 (82.6)	35 (59.3)
Having as much information as needed^a^	54 (65.9)	17 (73.9)	37 (62.7)
Sleeping difficulties^a^	53 (64.6)	12 (52.2)	41 (69.5)
Nausea and/or vomiting^a^	52 (63.4)	10 (43.5)	42 (71.2)
Having psychological needs met^a^	49 (59.8)	16 (69.6)	33 (55.9)
Having social support needs addressed^a^	48 (58.5)	18 (78.3)	30 (50.9)
Being able to access and undertake education^a^	48 (58.5)	11 (47.8)	37 (62.7)
Seizures^a^	45 (54.9)	10 (43.5)	35 (59.3)
Dystonia/muscle spasm^a^	43 (52.4)	8 (34.8)	35 (59.3)
Changes to physical function^a^	42 (51.2)	8 (34.8)	34 (57.6)
Setting and achieving life goals^a^	40 (48.8)	13 (56.5)	27 (45.8)
Financial burden of care^a^	38 (46.3)	19 (82.6)	19 (32.2)
Agitation^a^	37 (45.1)	4 (17.4)	33 (55.9)
Bowel problems^a^	37 (45.1)	6 (26.1)	31 (52.5)
Changes to appetite and/or eating	33 (40.2)	7 (30.4)	26 (44.1)
Changes in physical appearance	27 (32.9)	3 (13.0)	24 (40.7)
Having spiritual needs met	26 (31.7)	2 (8.7)	24 (40.7)
Changes in behaviour	25 (30.5)	9 (39.1)	16 (27.1)
Infections and/or impaired immunity^a^	25 (30.5)	12 (52.2)	13 (27.1)
Impact of illness on cognition	24 (29.3)	9 (39.1)	15 (25.4)
Having cultural needs addressed	21 (25.6)	0	21 (35.6)
Having religious and faith needs met	16 (19.5)	0	16 (27.1)
Cough	16 (19.5)	3 (13.0)	13 (22.0)
Changes in consciousness	15 (18.3)	3 (13.0)	12 (20.3)
Changes to self-outlook	14 (17.1)	5 (21.7)	9 (15.3)
Skin concerns	13 (15.9)	4 (17.4)	9 (15.3)
Weight changes	10 (12.2)	5 (21.7)	5 (8.5)
Opportunity to explore the meaning of life	9 (11.0)	4 (17.4)	5 (8.5)
Being able to leave a legacy	6 (7.3)	4 (17.4)	2 (3.4)
Low blood counts	5 (6.1)	4 (17.4)	1 (1.7)
Fertility concerns	4 (4.9)	1 (0.2)	3 (5.1)

a Items moved to ranking rounds (*n* = 27). ^b^HSCP = health and social care professional.

#### Round 2–‘Ranking’ round phase i

Sixty individuals (47 professionals; 13 parents) participated in ranking the 27 retained items. See supplementary Table 2 for demographics. There was weak overall agreement on ranking (*W* = 0.12). There was also weak agreement between parents’ rankings alone (*W* = 0.16) and professionals alone (*W* = 0.21). Cohen’s kappa between parents and professionals was 0.08 (Table 3).

**Table 3.** Delphi results round 2 – ranking phase I.

Outcome (*n* = 27)	Overall median rank (% ranking in top 50%) (*n* = 60)	Parent median rank (% ranking in top 50%) (*n* = 13)	HSCP median ranking (% ranking in top 50%) (*n* = 47)
Pain	5.5 (88.3)	7 (84.6)	1 (89.4)
Ability to live life to the fullest	6.5 (66.7)	5 (76.9)	5 (63.8)
Breathing and respiratory difficulties	7 (80.0)	12 (69.2)	2 (83.0)
Child/young person being able to do things they enjoy	8 (73.3)	6 (69.2)	3 (74.5)
Having sufficient support from health and social care professionals	9 (68.3)	9 (76.9)	6 (66.0)
Having a plan for future care	9.5 (68.3)	14 (61.5)	4 (70.2)
Dystonia/muscle spasms	11.5(60.0)	18 (38.5)	9 (66.0)
Being supported/enabled to express emotions and feelings	12 (58.3)	11 (53.8)	10 (59.8)
Sleeping difficulties	12.5 (58.3)	12 (76.9)	12 (53.2)
Setting and achieving life goals	12.5(50.0)	13 (53.8)	19 (48.9)
Having psychological needs met	12.5 (53.3)	9 (61.5)	16 (51.1)
Nausea and vomiting	13 (58.3)	19 (23.1)	7 (68.1)
Tiredness or fatigue	13.5 (56.7)	14 (61.5)	11 (55.3)
Reducing the impact of illness on family life/care burden	13.5 (53.3)	14 (53.8)	15 (53.2)
Emotional impact of illness	14 (55.0)	11 (53.8)	14 (55.5)
Seizures	14 (56.7)	14 (46.1)	8 (59.6)
Agitation	15.5 (51.2)	20 (15.4)	13 (61.7)
Siblings being supported and having their needs met	16(38.3)	14 (61.5)	21 (31.9)
Changes to physical function	16.5 (41.2)	14 (53.8)	20 (38.3)
Bowel problems	17 (43.3)	19 (23.1)	18 (48.9)
Having as much information as needed	17 (48.3)	17 (46.2)	17 (48.9)
Being able to maintain relationships with peers	18 (36.7)	15 (46.2)	23 (34.0)
Being able to take part in memory making opportunities	19.5 (33.3)	20 (30.8)	22 (34.0)
Financial burden of care	20 (25.0)	15 (46.2)	25 (19.1)
Infections and/or impaired immunity	20 (26.7)	19 (38.5)	24 (23.4)
Having social support needs addressed	20.5 (23.3)	17 (38.5)	26 (19.1)
Being able to access and undertake education	22.5 (26.7)	22 (38.5)	27 (59.6)
Kendall’s *W*	0.1671	0.1595	0.2053

#### Round 3 – ‘Ranking’ round phase ii

Thirty individuals participated in round 3 (26 professionals; 4 parents) and the 27 items ranked in the previous round were ranked again. See supplementary Table 3 for demographics. Overall, agreement between participants was moderate (*W* = 0.61). There was also moderate agreement between the professional group alone (*W* = 0.68) and parent group alone (*W* = 0.64). Cohen’s kappa between parent and professionals =0.13 (poor agreement) (Table 4).

**Table 4.** Delphi results round 3 – ‘ranking’ round phase II.

Outcome	Overall median rank (% ranking in top 50%) (*n* = 30)	Parent median rank (% ranking in top 50%) (*n* = 4)	HSCP median rank (% ranking in top 50%) (*n* = 26)	Times item identified in top 5 during involvement activity with older children and young people (11 representatives)	Item identified in overall top 13 during involvement activity with younger children (11 representatives)
Pain	1 (90.0)	9.5 (50.0)	1 (96.2)	7	Yes
Ability to live life to the fullest	2 (96.7)	1.5 (100)	2.5 (96.2)	3	Yes
Breathing and respiratory difficulties	3 (96.7)	6.5 (100)	3 (96.2)	2	Yes
Child/young person being able to do things they enjoy	4 (96.7)	4 (100)	4 (96.2)	5	Yes
Having sufficient support from HSCPs	5 (93.3)	5.5 (75)	5 (92.3)	3	No
Having a plan for future care	6 (90.0)	9.5 (25)	6 (92.3)	1	No
Dystonia/muscle spasms	8 (76.7)	20 (25)	7 (84.6)	0	No
Being supported/enabled to express emotions and feelings	9 (80.0)	8 (100)	9.5 (76.9)	2	No
Sleeping difficulties	10.5 (86.7)	10.5 (75)	10.5 (88.5)	3	No
Having psychological needs met	10.5 (76.7)	9.5 (100)	11 (73.1)	5	No
Nausea and vomiting	12 (76.7)	17 (50)	11.5 (80.8)	1	Yes
Setting and achieving life goals	12 (73.3)	8.5 (100)	12 (69.2)	1	No
Tiredness or fatigue	13 (80.0)	5.5 (100)	13 (76.9)	3	No
Reducing the impact of illness on family life/care burden	14.5 (50.0)	13.5 (75)	15.5 (46.2)	2	Yes
Agitation	16 (36.7)	20 (0)	16 (42.3)	0	No
Seizures	16 (36.7)	16.5 (0)	16 (42.3)	0	Yes
Emotional impact of illness	16 (23.3)	10.5 (75)	17 (15.4)	3	Yes
Siblings being supported and having their needs met	18 (16.7)	22 (0)	18 (19.2)	0	No
Changes to physical function	19 (16.7)	10.5 (75)	19 (7.7)	2	Yes
Having as much information as needed	20 (13.3)	20.5 (0)	20 (15.4)	0	No
Bowel problems	20.5 (23.3)	20.5 (0)	20.5 (26.9)	0	No
Being able to maintain relationships with peers	22 (13.3)	22 (0)	22 (15.4)	5	Yes
Being able to take part in memory making opportunities	23 (13.3)	16 (25)	23 (11.5)	0	Yes
Infections and/or impaired immunity	24 (6.7)	20.5 (25)	24 (3.8)	1	No
Financial burden of care	25 (20.0)	24.5 (25)	25 (19.2)	0	No
Having social support needs addressed	26 (10.0)	21.5 (25)	26 (7.7)	0	No
Being able to access and undertake education	27 (6.7)	23.5 (0)	27 (7.7)	1	Yes
Kendall’s *W*	*W* = 0.61	*W* = 0.68	*W* = 0.64	–	–

As Kendall’s W had increased from weak to moderate agreement the decision was taken to stop the study at this point due to concerns regarding potential gain and feasibility of conducting another round.

### Consultation with Young Person’s Advisory Group

Twenty-two children (17 female; 6 male) aged 10–21 years attended the meeting. The responses given by two groups are shown in Table 4. Both groups suggested naming the C-POS versions after planets to avoid any stigma using chronological age. Measure selection will be dependent on developmental stage.

#### Phase 2 – Item generation meeting

Twenty-two members attended the item generation meeting – nine paediatric palliative care clinicians, six research team members, five clinical academics with expertise in PCOM development and two bereaved parents. After the initial presentations, each domain from our qualitative interview study was discussed and potential C-POS items were mapped onto these.^6,36^ Previous work had suggested children’s care priorities differed from parents, particularly regarding practical aspects of care. It was agreed that C-POS would have self-report items regarding children’s symptoms and concerns, and separate questions for parents to answer regarding family concerns.^6^ It was further agreed that there would be proxy versions of the measure for parents to answer on behalf of their child if they were unable to respond themselves. Proxy versions would contain the same items as the self-report versions.

Five versions of the measure were drafted, each with eight questions about the child and five about the family: (1) parent/carer of child<2 years, (2) parent/carer of child ⩾2 years, (3) child 5–7, (4) 8–12 and (5) 13–18 years (or cognitive equivalent). The number of items was informed by previous work which suggested that children should have 10 items or fewer to respond to.^24^ These versions were named after planets, as suggested by the young person’s advisory group. Items were the same across versions but were worded differently in consideration of age/developmental stage. For example, using the term ‘hurt’ rather than ‘pain’. Recall period and response format were based on previous evidence, with shorter recall and a three-point Likert scale for younger/less cognitively able children, and a longer recall and five-point Likert scale for older/more cognitively able children.^23,24^ The Likert scales on the child versions were anchored with emojis. Table 5 shows domains and agreed items for C-POS.

**Table 5.** Mapping of C-POS items onto domains from previous qualitative interview study and systematic review.^6,36^

Child symptom and concern items (self-reported or proxy-reported)	
Domain	Question item
Physical	Pain
	Other symptoms
Social and practical	Being able to ask questions
	Being able to undertake usual activities
Emotional and psychological	Worry
	Sharing feelings
	Being able to do things you enjoy
Spiritual/existential	Being able to do things you enjoy
	Living life to the fullest
Parent/carer items	
Physical	Getting enough sleep
Social/practical	Access to information about child’s condition
	Support needed to care for child
	Support to plan for future care
Emotional/psychological	Impact of child’s condition on family
Spiritual/existential	Support to plan future care

Due to the number and heterogeneity of life-limiting conditions,^37^ ensuring suitability of all items for the entire population proved challenging. Several physical symptoms (e.g., dystonia and breathing difficulties) were prioritised in the Delphi survey, but not all children with life-limiting conditions experience these. Only pain was common across the population. Hence a decision was taken to have a generic question regarding symptoms other than pain. The item regarding siblings was not relevant to all families, so a question regarding the impact of the child’s condition on the family was worded to incorporate relevant family members.

## Discussion

This paper reports on the development of the first parent-proxy and age/developmental stage appropriate child versions of an outcome measure for children with life-limiting conditions and their families outside of sub-Saharan Africa.^21,27^ The Delphi survey, young person’s advisory group, and item generation meeting have together established face and content validity of the proposed C-POS. This research ensures that the proposed items to undergo further psychometric testing reflect the construct we intend to measure, i.e., priority multidimensional palliative care outcomes for children with a range of life-limiting conditions, their families and the professionals caring for them. Importantly, C-POS items capture all domains covered in the World Health Organisation’s definition of paediatric palliative care.^38^

Parent and professional Delphi rankings contained many similarities, but there were some differences, resulting in low inter-relater reliability between the two groups. Professionals were more likely to prioritise physical symptoms such as pain, respiratory difficulties and dystonia. Parents were more likely to prioritise psychosocial concerns such as memory making and the emotional impact of a life-limiting condition. Parents were also more likely to prioritise their child’s physical function, possibly because these impact family care burden as well as participation in activities outside the home, some of which are important to siblings. While many elements of palliative care are important to both professionals and parents,^39^ some studies indicate that professionals put greater emphasis on physical well-being.^40^ The final C-POS versions address these differences by incorporating items that were highlighted as a priority by either and both stakeholder groups.

Consultation with members of the young person’s advisory group identified similarities between the Delphi results and the selection of priority items by adult participants, particularly in relation to managing physical symptoms such as pain, being able to live life to the fullest and undertake activities that provide enjoyment. However, the group also identified the importance of being able to access education and maintain peer relations. These items were not ranked in the top 50% by parents or professionals. This finding corroborates previous research that identified the importance of addressing not only physical needs but also supporting pursuit of activities which are part of normalcy for children.^6,41–43^ Input from the group informed the C-POS item regarding ability to undertake usual activities. It also highlights the importance of input from all stakeholder groups in the development of PCOMs. The involvement of children and young people affirms that it is both possible and vital for children to have the opportunity to participate in the development of PCOMs intended for their use, and not rely on proxy reporting alone.^44,45^

### What this study adds

Our robust, sequential approach to the development of C-POS has ensured that items are an accurate reflection of the outcomes that are important to children with life-limiting conditions and their families.^26^ Involving professionals in the measure development process has helped raise awareness of the development of C-POS and the use of PCOMs in clinical practice. Evidence shows that healthcare professionals need more education on the use and implementation of PCOMs in clinical practice, and suggests that engaging professionals in measure development processes should help to achieve this.^46^

### Strengths and limitations

The C-POS development process follows outcome measure development guidance from COSMIN and Rothrock.^21,26^ This has ensured that by involving key stakeholders C-POS has excellent face and content validity for the construct being measured, the target population and context of use.^27^ Delphi participants were recruited from across three of the four UK nations, and from multiple regions in England. There is geographical variation in UK paediatric palliative care service provision, and widespread recruitment allowed for differences in priority based on provision to be accounted for.^5^ We recruited a relatively large number of participants, with many Delphi surveys recruiting less than 50 participants.^47^

The lack of ethnic diversity of parents recruited to the Delphi survey is not reflective of the population of children who require palliative care in the UK. Those from Asian, Black and Bangladeshi backgrounds are more likely to have life-limiting conditions.^4^ Our parent participants all identified as white British, with four saying their child was of mixed ethnic group. Future research should focus on ways to increase ethnic diversity in paediatric palliative care research, and we will seek to recruit participants from minoritised groups in future C-POS validation work. All of our parent participants were female and this is consistent with much of paediatric palliative care research, i.e. fathers are often under-represented.^48^

By round 3 of the Delphi survey only 36.5% of original participants responded. This attrition rate is similar to other Delphi surveys in paediatric palliative care where parents and professionals were included as participants.^16^ In our study, attrition was particularly high in parents, with parents forming 15% of the sample in round 3. This can be attributed to two national COVID-19 pandemic lockdowns during recruitment. These lockdowns led to loss of vital social support and disruption to essential healthcare services, placing additional care burden on families of children with life-limiting conditions.^49^ As a result of attrition and concerns about the feasibility of a further round and potential gain, it was decided to stop the Delphi survey before reaching the predetermined criteria (*W* > 0.7).^29^ There is no uniform definition for consensus in Delphi surveys. Although achieving *W* > 0.7 is often used as a stopping criterion, most ranking-type Delphi’s report a moderate final consensus rate (*W* = 0.5–0.7).^30,47^ Our Kendall’s *W* coefficient of concordance increased from weak to moderate between rounds 2 and 3, suggesting a move towards consensus. The increase in proportion of health care professionals in the final ranking round could potentially have contributed to this increase in consensus.

### Next steps

Further research is required to demonstrate the comprehensiveness, comprehensibility and acceptability of C-POS using cognitive interviews, followed by psychometric testing.

## Conclusions

C-POS has undergone a robust development process using accepted methodological guidance on PROM development. This has ensured items within the measure reflect the construct set out to be measured, and that they have face and content validity within the target population. Important differences were found in priority outcomes identified by different stakeholder groups, highlighting the importance of involving all key stakeholders in PCOM development.

## Supplemental Material

sj-pdf-1-pmj-10.1177_02692163231205126 – Supplemental material for Achieving consensus on priority items for paediatric palliative care outcome measurement: Results from a modified Delphi survey, engagement with a children’s research involvement group and expert item generation Click here for additional data file. Supplemental material, sj-pdf-1-pmj-10.1177_02692163231205126 for Achieving consensus on priority items for paediatric palliative care outcome measurement: Results from a modified Delphi survey, engagement with a children’s research involvement group and expert item generation by Lucy Coombes, Daney Harðardóttir, Debbie Braybrook, Hannah May Scott, Katherine Bristowe, Clare Ellis-Smith, Lorna K Fraser, Julia Downing, Myra Bluebond-Langner, Fliss EM Murtagh and Richard Harding in Palliative Medicine

sj-pdf-2-pmj-10.1177_02692163231205126 – Supplemental material for Achieving consensus on priority items for paediatric palliative care outcome measurement: Results from a modified Delphi survey, engagement with a children’s research involvement group and expert item generation Click here for additional data file. Supplemental material, sj-pdf-2-pmj-10.1177_02692163231205126 for Achieving consensus on priority items for paediatric palliative care outcome measurement: Results from a modified Delphi survey, engagement with a children’s research involvement group and expert item generation by Lucy Coombes, Daney Harðardóttir, Debbie Braybrook, Hannah May Scott, Katherine Bristowe, Clare Ellis-Smith, Lorna K Fraser, Julia Downing, Myra Bluebond-Langner, Fliss EM Murtagh and Richard Harding in Palliative Medicine
