# Supplementary material for: Achieving consensus on priority items for paediatric palliative care outcome measurement: Results from a modified Delphi survey, engagement with a children’s research involvement group and expert item generation
Source: Palliat Med. 2023 Oct 18;37(10):1509–19. doi: 10.1177/02692163231205126 (PMC10657511; doi:10.1177/02692163231205126)
Supplement: sj-pdf-1-pmj-10.1177_02692163231205126 – Supplemental material for Achieving consensus on priority items for paediatric palliative care outcome measurement: Results from a modified Delphi survey, engagement with a children’s research involvement group and expert item generation [file sj-pdf-1-pmj-10.1177_02692163231205126.pdf]

Supplementary file 1 – Round 2 Delphi survey participant demographics

| **Health and social care professionals (n=47)** | | **Parent/carers (n=13)** | |
|---|---|---|---|
| **Gender (male:female)** | 6:41 | **Gender (male:female)** | 0:13 |
| **Profession** | 11 Doctor<br>28 Nurse<br>1 Physiotherapist<br>2 Health care assistant<br>5 Counsellor/therapist | **Child's diagnosis** | 4 Metabolic<br>5 Congenital<br>3 Neurological<br>1 Genitourinary |
| **Place of work** | 15 Hospital<br>25 Hospice<br>4 Community<br>3 Multiple settings | **Child's age years (mean; range)** | 9.3 (1-16) |
| **UK region** | 5 England-Northeast<br>4 England – Southeast<br>4 England – Southwest<br>3 England – West Midlands<br>3 England – Yorkshire and Humber<br>22 England – East<br>2 Wales<br>2 England – East Midlands<br>2 England – Northwest | **UK region** | 4 England – Southeast<br>2 England – Yorkshire and Humber<br>6 England – East<br>1 Scotland |
| **Experience years (mean; range)** | 13.2; 1-36 | **Ethnic background** | 13 white British (parent/carer)<br>3 mixed ethnic group: 10 white British (child) |
