# Supplementary material for: Achieving consensus on priority items for paediatric palliative care outcome measurement: Results from a modified Delphi survey, engagement with a children’s research involvement group and expert item generation
Source: Palliat Med. 2023 Oct 18;37(10):1509–19. doi: 10.1177/02692163231205126 (PMC10657511; doi:10.1177/02692163231205126)
Supplement: sj-pdf-2-pmj-10.1177_02692163231205126 – Supplemental material for Achieving consensus on priority items for paediatric palliative care outcome measurement: Results from a modified Delphi survey, engagement with a children’s research involvement group and expert item generation [file sj-pdf-2-pmj-10.1177_02692163231205126.pdf]

Supplementary File 2 - Round 3 Delphi survey participant demographics

**Health and social care professionals (n=26)**		**Parent/carers (n=4)**	
**Gender (male:female)**		**Gender (male:female)**	0:4
**Profession**	9 Doctor<br>14 Nurse<br>1 Physiotherapist<br>1 Health care assistant<br>1 Counsellor/therapist	**Child's diagnosis**	1 Metabolic<br>1 Congenital<br>1 Neurological<br>1 genitourinary
**Place of work**	9 Hospital<br>11 Hospice<br>4 Community<br>2 Multiple settings	**Child's age years (mean; range)**	12.0 (2-16)
**UK region**	3 England-Northeast<br>2 England – Southeast<br>1 England – Southwest<br>2 England – West Midlands<br>1 England – Yorkshire and Humber<br>12 England – East<br>2 Wales<br>2 England – East Midlands<br>1 Greater London	**UK region**	1 England – Southeast<br>1 England – Yorkshire and Humber<br>2 England – East
**Experience years (mean; range)**	13.3; 1.5-36	**Ethnic background**	4 white British (parent/carer)<br>1 mixed ethnic group: 3 white British (child)
